# Charge Regulation of Poly(acrylic acid) in Solutions of Non-Charged Polymer and Colloids

**DOI:** 10.3390/polym15051121

**Published:** 2023-02-23

**Authors:** Evgenee Yekymov, David Attia, Yael Levi-Kalisman, Ronit Bitton, Rachel Yerushalmi-Rozen

**Affiliations:** 1 Department of Chemical Engineering, Ben-Gurion University of the Negev, Beer-Sheva 84105, Israel; 2 The Center for Nanoscience and Nanotechnology, The Institute of Life Sciences, The Hebrew University, Jerusalem 91904, Israel; 3 The Ilse Katz Institute for Nanoscience and Technology, Ben-Gurion University of the Negev, Beer-Sheva 84105, Israel

**Keywords:** poly(acrylic acid), weak polyelectrolytes (WPEs), acid–base, equilibrium, *pK_a_*, *pH*, excluded volume, depletion interactions, crowding, steric effects

## Abstract

Weak polyelectrolytes (WPEs) are responsive materials used as active charge regulators in a variety of applications, including controlled release and drug delivery in crowded bio-related and synthetic environments. In these environments, high concentrations of solvated molecules, nanostructures, and molecular assemblies are ubiquitous. Here, we investigated the effect of high concentrations of non-adsorbing, short chains of poly(vinyl alcohol), PVA, and colloids dispersed by the very same polymers on charge regulation (CR) of poly(acrylic acid), PAA. PVA does not interact with PAA (throughout the full pH range) and thus can be used to examine the role of non-specific (entropic) interactions in polymer-rich environments. Titration experiments of PAA (mainly 100 kDa in dilute solutions, no added salt) were carried out in high concentrations of PVA (13–23 kDa, 5–15 wt%) and dispersions of carbon black (CB) decorated by the same PVA (CB-PVA, 0.2–1 wt%). The calculated equilibrium constant (and $pK_{a}$) was up-shifted in PVA solutions by up to ~0.9 units and down-shifted in CB-PVA dispersions by ~0.4 units. Thus, while solvated PVA chains increase the charging of the PAA chains, as compared to PAA in water, CB-PVA particles reduce PAA charging. To investigate the origins of the effect, we analyzed the mixtures using small-angle X-ray scattering (SAXS) and cryo-TEM imaging. The scattering experiments revealed re-organization of the PAA chains in the presence of the solvated PVA but not in the CB-PVA dispersions. These observations clearly indicate that the acid–base equilibrium and the degree of ionization of PAA in crowded liquid environments is affected by the concentration, size, and geometry of seemingly non-interacting additives, probably due to depletion and excluded volume interactions. Thus, entropic effects that do not depend on specific interactions should be taken into consideration when designing functional materials in complex fluid environments.

## 1. Introduction

Flexible polymers comprising weak polyacids (such as poly(acrylic acid), PAA) are responsive materials as the acid–base reaction: $HA\;⇄H^{+}+A^{-}$ of the covalently linked groups is not only determined by the bath *pH* and ionic strength but also by the polymer’s ability to regulate the charge (CR) in response to a variety of external conditions [1,2,3].

The degree of ionization α, of simple acid in an ideal bulk solution, where all the reacting species are mobile, is:

(1) $$\alpha =\frac{\left[A^{-}\right]}{\left[AH\right]+\left[A^{-}\right]}=\frac{1}{1+10^{pK_{a}-pH}}$$

pH is the bulk *pH*, $K_{a}$ is the equilibrium constant of the dissociation reaction, and $pK_{a}$ is (−log K_a_) [4].

In solutions of weak polyelectrolytes (WPEs), $pK_{a}$ is no longer constant (at a given temperature) but varies along the titration curve. In their seminal work, Katchalsky and coworkers [5,6] suggested that in dilute solutions, the degree of ionization of a fully hydrophilic polymer (such as PAA) would vary linearly with $\alpha^{\frac{1}{3}}$:

(2) $$pH=pK_{a}-log\left(\frac{1-\alpha }{\alpha }\right)+0.4343.2\lambda {\left(\frac{3}{\lambda^{\prime }sj}\right)}^{\frac{1}{3}}{\left(\frac{\varepsilon^{2}}{Dk_{b}Tb}\right)}^{\frac{2}{3}}\alpha^{\frac{1}{3}}$$

where $pK_{a}$ is $-log\left(K_{a}\right)$ of the monomers, *s* is the number of monomers, *b* is the length of the statistical unit (Kuhn length), *j* is the number of monomers per acid group, ε is the electron charge, D is the solvent dielectric constant, and $k_{b}T$ is the Boltzmann constant and absolute temperature. λ and $\lambda^{\prime }$ are parameters that depend on the extension of the coil. They are known to change slowly during titration [7]. The simplified equation is often used to fit experimental data:

(3) $$pH=pK_{a}-log\left(\frac{1-\alpha }{\alpha }\right)+const\alpha^{\frac{1}{3}}$$

Thus, the theory predicts that linear WPE chains in dilute solutions would become weaker acids along the titration curve as $pK_{a}$ increases with the increasing degree of ionization.

Additional complications arise when WPE chains are solvated in crowded environments, such as biological media or synthetic complex fluids where macromolecules and molecular assemblies can occupy up to 20–40% of the total volume [8,9]. Fundamental studies of WPE properties and responsiveness in concentrated polymer solutions and colloidal dispersions can highlight the role of coupling between the ionization of the WPEs and their interaction with the additional components via specific intermolecular interactions, conformational entropy of the chains [10], and excluded volume (steric) interactions [1,2,3].

Here we designed and characterized a model system dominated by macromolecular crowding and depletion interactions induced by high concentrations of a non-ionic polymer and colloidal particles. Dilute (0.1 and 1 wt%) solutions of PAA, with no added salt, and high concentrations (5–15 wt%) of a non-ionic highly hydrolyzed (98%) poly (vinyl alcohol), PVA, or PVA-decorated-Carbon Black particles (CB-PVA, typical single-particle diameter of 50–60 nm) were investigated. Highly hydrated PVA (98%, $M_{w}$ = 13–23 kDa) was chosen for this study, as isothermal titration calorimetry (ITC) measurements indicate that the intermolecular interactions between PAA and PVA are weak (0.03 kJ/mol [11]) and *pH*-independent. The PVA-decorated CB system provides a colloidal additive with high excluded volume compared to the solvated PVA chains.

Surprisingly, we found that while high concentrations (≤15 wt%) of PVA increased the degree of ionization (and reduced the $pK_{a}$) of PAA compared to PVA-free solutions (at the same concentration of PAA, no added salt), PVA-decorated CB (concentrations ≤ 1 wt%) reduced the degree of ionization of PAA (and increased the $pK_{a}$).

These results are discussed in the context of a previous study by us [10], reporting the effect of coupling between hydrogen bonding and excluded volume interactions on the titration curves and $pK_{a}$ of PAA in micellar solutions (of PEO-PPO-PEO (Pluronics) triblock copolymers and Brij-S20). Titration experiments of dilute, salt-free solutions of PAA indicated coupling of hydrogen bonding (between the protonated carboxylic group of PAA and the etheric oxygen of the PEO at the low *pH* regime) and excluded volume interactions resulting in a reduced degree of ionization of the PAA, consequentially increasing $pK_{a}$ (by up to ~0.7 units) in the acidic regime of the titration curve. 

The findings presented in the current study highlight the effect of coupling between steric (entropic) interactions and the acid–base reaction of WPEs, leading to CR and modification of the $pK_{a}$.

## 2. Experimental

### 2.1. Materials

Propionic acid (PAc) ~ 99.5 wt% was purchased from Sigma Aldrich, Rechovot, Israel (product #402907). The reported $pK_{a}$ is 4.87–4.9 [12,13,14].

**Sketch 1:**
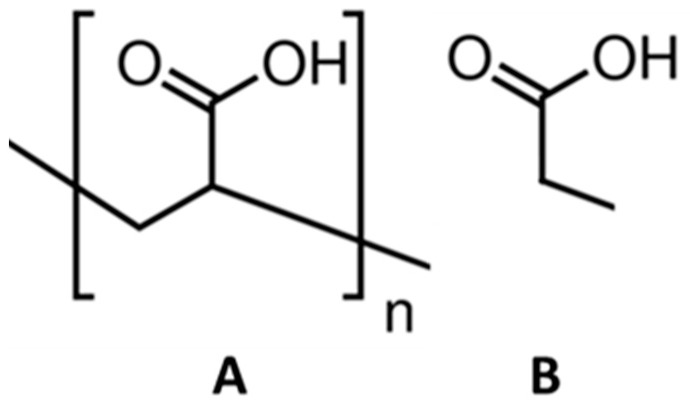
Chemical structure of (**A**) polyacrylic acid, (**B**) propionic acid.

PAA of different molecular weights (Table 1) was purchased as aqueous solutions of 30 kDa (30 wt%) (product #24771, Polysciences Inc., Warrington, PA, USA) and 100 kDa (35 wt%) (product #523925, Sigma Aldrich, Rechovot, Israel,). The $M_{w}$, the number of Khun segments (N), the hydrodynamic radius (R_H_), the measured overlap concentration (C*), and the (measured) $pK_{a}$ of PAA at the degree of ionization α = 0.5 are presented in Table 1. 

Poly (vinyl alcohol), PVA, $M_{w}$ 13–23 kDa, 98% hydrolysis was purchased from Sigma Aldrich, Rechovot, Israel (product # 363170).

HCl standard solution of 1 N and 0.1 N (product #H9892 and #2104, Sigma Aldrich, Rechovot, Israel ) and NaOH solutions 1 N and 0.1 N (product # 2770 and # 43617, Sigma Aldrich, Rechovot, Israel) were used following calibration. Carbon black nanopowder, d < 500 nm, was purchased from Sigma Aldrich, Rechovot, Israel (product #699632).

### 2.2. Preparation of Solutions

PAA solutions were prepared by mixing PAA stock solution with deionized water (DIW, Millipore water (18 MΩ×m)) or the relevant solutions (dispersions) to a final concentration.

PVA solutions were prepared by stirring the polymer powder in DIW for 5 h at 80 °C for complete dissolution. The solutions were stored at 60 °C for several days.

CB dispersions were prepared by sonicating the powder (5 wt%) in solutions of PVA (5 wt%) (sonication bath, 52 W) for 1 h. The resulting dispersions were filtered using Corning® 500 mL Vacuum Filter (0.22 µm Pore 33.2 cm^2^ PES Membrane). The dispersions were washed in DIW, after which the powder was dried at room temperature and re-dispersed in DIW.

### 2.3. Characterization

#### 2.3.1. Automatic Potentiometric Titration 

Titrations were performed at 22 ±1 °C using a homemade auto-titrator based on an Orion star a214 *pH* meter equipped with a METER TOLEDO glass *pH* electrode, with a ceramic junction ARGENTHAL™ Ag+-trap (Ag/AgCl) and 3 mol/L KCl reference electrolyte. The *pH* meter was calibrated using standard buffers with *pH* = 4.01, 7, and 10.04. Automatic titration was carried out using a weight-calibrated syringe pump (NE-1000, New Era Pump Systems Inc., Farmingdale, NY, USA) and controlled using a computer.

The following sequence was used in the measurements: the *pH* of the unstirred solution (15 mL) was measured; then, 20–35 μL of titrant (NaOH or HCl) was injected and stirred for 45 s, allowed to rest for 90 s, and re-measured. 

#### 2.3.2. Small-Angle X-ray Scattering (SAXS)

Scattering patterns of the solutions were collected using a SAXSLAB GANESHA 300-XL Xenocs, Grenoble, France. CuΚα radiation was generated by a Genix 3D Cu-source with an integrated monochromator, 3-pinhole collimation, and a two-dimensional Pilatus 300 K detector. The scattering intensity I(q) was recorded in the interval of 0.007 < q < 0.25 Å^−1^ (corresponding to the length scale of 25–900 Å), where the scattering vector is defined as $q=\left(4\pi /\lambda \right)\times \sin\theta$*,* with 2*θ* and *λ* being the scattering angle and wavelength, respectively. The measurements were performed under vacuum at an ambient temperature (~22 °C). The solutions were sealed in thin-walled quartz capillaries about 1.5 mm in diameter and 0.01 mm wall thickness; the scattering curves were corrected for counting time and sample absorption. The 2D SAXS patterns were azimuthally averaged to produce one-dimensional intensity profiles, I vs. q, using the two-dimensional data reduction program SAXSGUI. 

Some of the SAXS measurements were performed at the B21 beamline with the Diamond light source synchrotron, located at the Harwell Science and Innovation Campus in Oxfordshire, UK. An energy of 13.1 keV corresponding to a wavelength of 0.9464 A^−1^ was selected. The scattering intensity was recorded using an EigerX 4M (Dectris) detector in the interval 0.0045 < q < 0.34 A^−1^. 

The scattering spectra of the solvent were subtracted from the corresponding solution data using the Irena package [15] in Igor Pro 9 from WaveMetrics (Portland, Oregon) for the analysis of small-angle scattering data. Data analysis was based on fitting the scattering curve to an appropriate model using the SasView program [16].

#### 2.3.3. Transmission Electron Microscopy (TEM)

Rapid cooling enables the direct imaging of molecular assemblies and nanostructures in aqueous media. The samples were prepared by applying a 3 μL drop to a TEM grid (300 mesh Cu Lacey substrate, Ted Pella, Ltd., Redding, CA, USA) following a short pre-treatment of the grid via glow discharge. The excess liquid was blotted, and the specimen was vitrified by rapid plunging into liquid ethane precooled by liquid nitrogen using a vitrification robot system (Vitrobot mark IV, FEI). The rapid cooling resulted in the physical fixation of the liquid state so as to preserve the native structures. Thus, it allowed examination of the polymeric assemblies in the high vacuum of the electron microscope at cryogenic temperature, which prevented the formation of either cubic or hexagonal ice. The vitrified samples were examined at −177 °C using a FEI Tecnai 12 G2 TWIN TEM operated at 120 kV and equipped with a Gatan model 626 cold stage. The images were recorded using a 4 K × 4 K FEI Eagle CCD camera in low-dose mode. TIA (Tecnai Imaging & Analysis) software was used to record the images.

## 3. Results

Potentiometric titrations of dilute solutions of PAA (0.1 wt% and 1 wt%, no added salt, see Table 1) of different molecular weights were carried out, followed by titrations of PAA in concentrated PVA solutions (5–13 wt%). The PVA used in this study ($M_{w}$ 13–23 kDa) was of lower molecular weight than the PAA (mainly 100 kDa) and was highly hydrolyzed (98%). Rheological measurements (ESI Figure S3) show Newtonian behavior up to 14 wt%. Thus, the measured solutions are below the overlap concentration or just above it (15 wt%) [17]. In addition, PVA was used for the preparation of CB dispersions. The dispersions were filtered to remove excess PVA, dried, and re-dispersed (see the experimental part), resulting in a dispersion of CB decorated by PVA (CB-PVA). Titration curves of PAA in solutions of PVA and dispersions of CB-PVA are presented below. 

### 3.1. Titration Experiments

**Figure 1:**
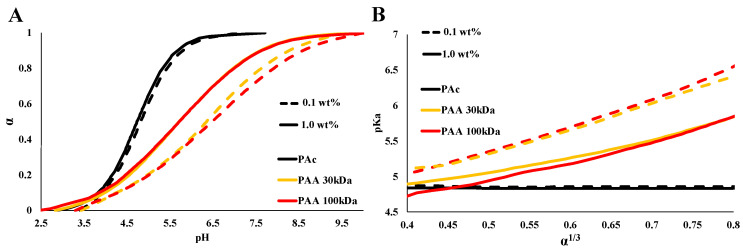
(**A**) Titration curves presenting the degree of ionization (α) as a function of *pH*. PAc, PAA 30 kDa, and PAA 100 kDa (**B**) $pK_{a}$ as a function of α ^1/3^. For PAA 0.1 (dashed) and 1 wt% (solid).

Titration curves of PAA in DIW are presented in Figure 1 for two molecular weights of PAA (30 kDa and 100 kDa). When compared to the titration curves of PAc, the curves show the expected effect of chain connectivity: Due to CR [7], the degree of ionization of the polymers (at a given *pH*) is lower than that of a monomeric acid of similar composition and structure (Sketch 1), and the titration curves are broadened. The values of $pK_{a}$, calculated for different degrees of ionization, as a function of α^1/3^ are presented in Figure 1B. As predicted by Equation (3), the $pK_{a}$ of PAA varied linearly with α^1/3^ [7,18,19,20], while in these conditions, the $pK_{a}$ of the monomeric acid (PAc) did not depend on the degree of ionization. Note that along the titration curve, the $pK_{a}$ increases by 1.5 *pH* units for 0.1 wt% PAA, indicating a significant reduction in the concentration of the charged groups of PAA, as compared to PAc. For example, at *pH* = 4.5, about 33% of the solvated PAc groups are ionized, while only about 13% of the PAA (0.1 wt%). The deviation of the titration curves of PAA from that of PAc is larger for the lower PAA concentration (0.1 wt% as compared to 1 wt% of PAA).

The two molecular weights examined here (PAA 30 kDa and PAA 100 kDa) show similar behavior. Thus, the following experiments were carried out using only PAA 100 kDa.

**Figure 2:**
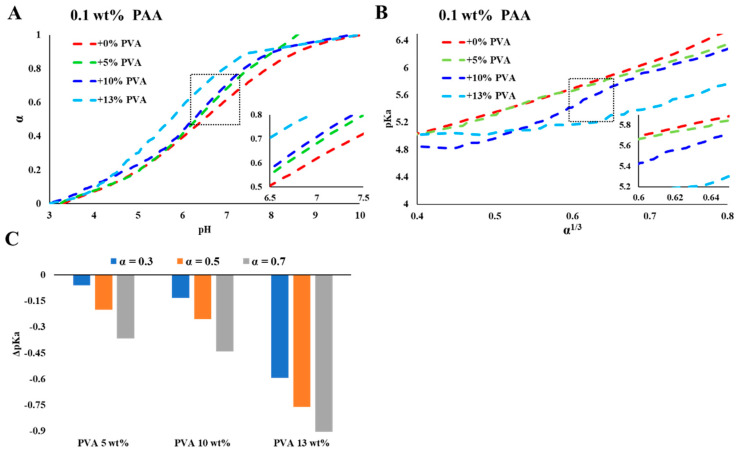
Titration curves of PAA 100 kDa (0.1 wt%) in PVA solutions. (**A**) The degree of ionization (α) as a function of *pH*, (**B**) $pK_{a}$ as a function of α ^1/3^, (**C**) Δ$pK_{a}$ (as compared to additive-free PAA solutions) in PVA solutions of 5, 10, and 13 wt% for α = 0.3, 0.5, and 0.7.

**Figure 3:**
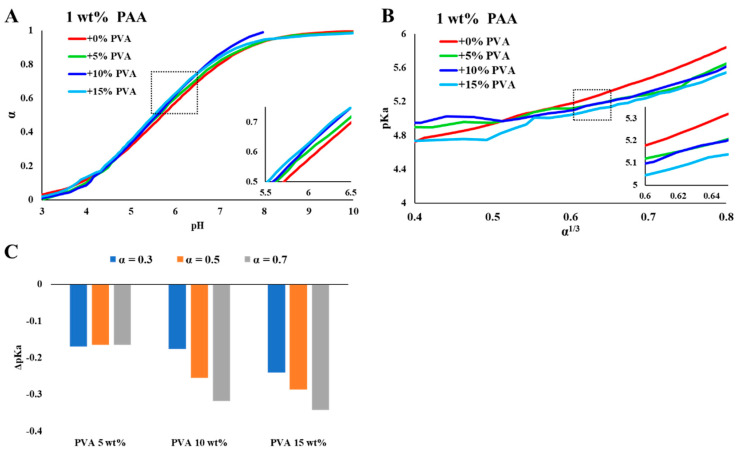
Titration curves of PAA 100 kDa (1 wt%) in PVA solutions. **(A)** The degree of ionization (α) as a function of *pH*, (**B**) $pK_{a}$ as a function of α ^1/3^, (**C**) Δ$pK_{a}$ (as compared to additive-free PAA solutions) in PVA solutions of 5, 10, and 15 wt%, for α = 0.3, 0.5, and 0.7.

In Figure 2 and Figure 3, we present the effect of high concentrations of PVA on the titration curves of PAA. The calculations of the degree of ionization and the $pK_{a}$ in PVA solutions are detailed in the ESI (Equations (S1)–(S6)). The curves indicate that the presence of solvated PVA chains increased the degree of ionization of PAA.

Titration curves of PAA (0.1 and 1 wt%) in concentrated PVA solutions (Figure 2A and Figure 3A) show a significant shift in the degree of ionization of PAA for a given *pH*, compared to PVA-free solutions. The calculated $pK_{a}$ values (presented as a function of α^1/3^, Figure 2B and Figure 3B) indicate that the degree of ionization of PAA increases due to the presence of PVA and depends on the concentration of PVA. The deviations (presented as Δ$pK_{a}$, Figure 2C and Figure 3C) increase with the concentration of PVA and are more significant for the lower PAA concentration (0.1 wt%, Figure 2).

### 3.2. SAXS Measurements of PAA in PVA Solutions

**Figure 4:**
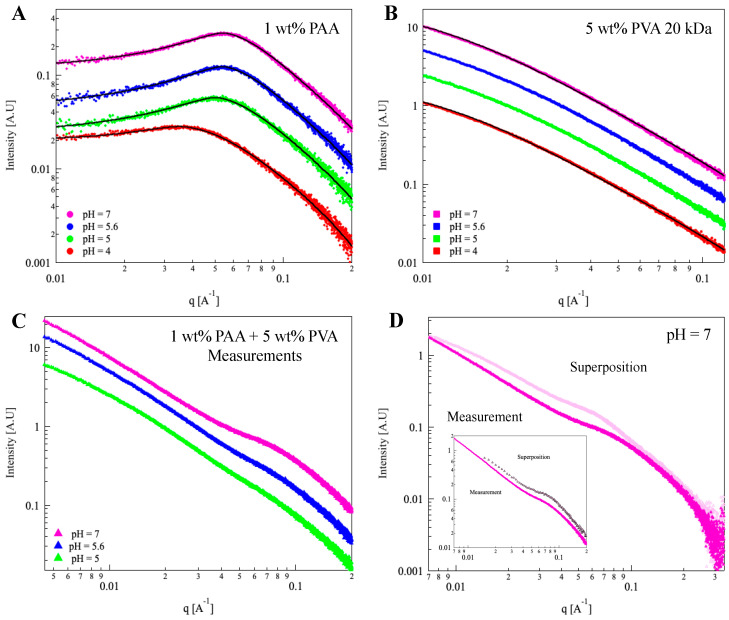
SAXS measurements. One-dimensional SAXS curves obtained from (**A**) 1 wt% PAA 100 kDa solutions, (**B**) 5 wt% PVA 20 kDa solutions, and (**C**) 1 wt% PAA-5 wt% PVA mixtures at *pH* = 4, 5, 5.6, 7. The solid black lines represent the best fit to Equations (S2)–(S4) (described in detail in the ESI) (**D**) Measured (▲) and calculated via superposition (▲) 1D SAXS curves obtained from 1 wt% PAA in 5 wt% PVA at *pH* = 7. Inset: SAXS curve obtained from 1 wt% PAA in 5 wt% PVA solution at *pH* = 7 (▲) and calculated curve via superposition of 1.6 wt% PAA and 5 wt% PVA at *pH* = 7 (▲). The curves are shifted for better visualization. Color bar: *pH* = 4 (▼), 5 (▼), 5.6 (▼), 7 (▼).

The nanostructure of PAA in PVA solutions was characterized using SAXS. As expected, the scattering curves of PAA and PVA solutions (Figure 4A,B) could be fitted to the *broad peak* and *Ornstein–Zernike* models, respectively (for additional details, see the ESI) [21,22].

The scattering curves of 1 wt% PAA in solutions of 5 wt% PVA at different *pH* values are presented in Figure 4C. All three curves exhibit a shoulder in the mid-q range (q ~ 0.07 A^−1^) that becomes more pronounced at higher *pH*. 

In Figure 4D (*pH* = 7), we present a scattering curve of the mixture alongside a curve derived by the superposition of the scattering curves of the components. The two curves differ in the low-mid q range, where the peak in the measured curve is shifted to higher q values. Interestingly, the linear superposition of a scattering curve of PVA (5 wt%) and a scattering curve measured in a solution of 1.6 wt% of PAA (instead of 1 wt%) enables us to reconstruct the measured curve of the mixture (Figure 4D inset, and Figure S7 of the ESI), indicating that the presence of PVA leads to a local crowding of the solvated PAA chains.

### 3.3. Titrations of PAA in CB-PVA Dispersions and Analysis of the Nanostructure of the Dispersions

Following the investigation of high concentrations of solvated PVA, we studied the effect of CB decorated by adsorbed PVA chains (PVA $M_{w}$ 13–23 kDa, 98% hydrolysis) on the CR in dilute PAA solutions. 

**Figure 5:**
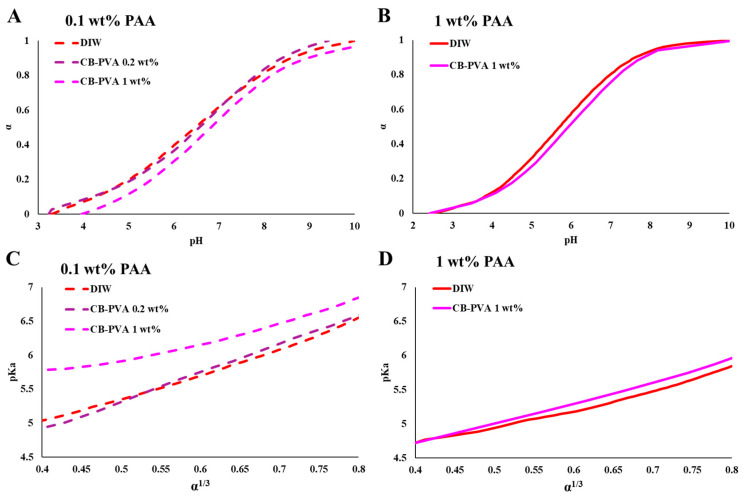
Titration curves presenting the degree of ionization (α) as a function of *pH* for PAA 100 kDa in dispersions of CB-PVA: (**A**) 0.1 wt% PAA, (**B**) 1 wt% PAA, (**C**) $pK_{a}$ as a function of α ^1/3^ in PAA (0.1 wt%) in CB-PVA dispersions, and (**D**) $pK_{a}$ as a function of α ^1/3^ in PAA (1 wt%) in CB-PVA dispersions.

In Figure 5, we present titrations curves of PAA 100 kDa (1 wt%) in PVA-decorated CB suspensions. The results show that the presence of dispersed CB-PVA shifts the titration curves. The calculated $pK_{a}$ values (presented as a function of α^1/3^, Figure 5C,D) indicate that the degree of ionization of PAA decreases in the dispersions compared to the PAA solutions in water (red curve in Figure 5C,D).

**Figure 6:**
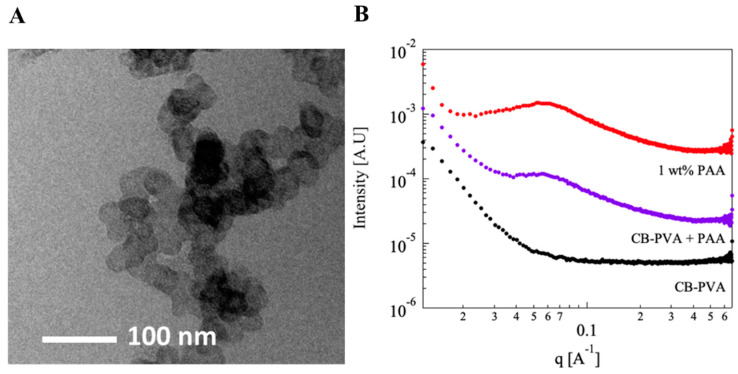
(**A**) Cryo-TEM image of a dispersion of 1 wt% CB -PVA at *pH* = 7 (see also an image of the dispersion at *pH* = 4, in Figure S4 of the ESI). (**B**) Raw 1D SAXS measurements of 1 wt% PAA (●) 0.2 wt% CB-PVA dispersion (●) and solvated 1 wt% PAA in 0.2 wt% CB-PVA dispersion (●), all at *pH* = 7. The curves are shifted for better visualization.

The cryo-TEM images of the CB-PVA dispersions presented in Figure 6A exhibit chain-like aggregates formed by the dispersed CB particles. Images taken at different *pH* values show similar behavior (see the ESI, Figure S4). Figure 6B presents 1D SAXS curves obtained from dispersions of 0.2 wt% CB-PVA in the presence of 1 wt% PAA at *pH* = 7. At the mid-q range, the SAXS curve of PVA-decorated CB dispersion (lower, black) exhibits the expected power low (*I ~ q^-n^*, *n* = −3.42) typical to a surface fractal (consistent with previous studies [23] and the cryo-TEM image). The SAXS curve obtained from CB dispersion in the presence of 1 wt% PAA curve (middle, purple) shows the characteristics of both entities, as evident in the peak at q ~ 0.057 A^−1^, which results from the presence of PAA, as observed in the curve obtained from 1 wt% solvated PAA solution at *pH* = 7 (top, red) and the upturn at the low-q range.

Superposition of the scattering curves obtained from each of the components, (Figure 6B) the lower curve (black) and upper curve (red), is identical to the scattering curve obtained by measuring the mixture (middle curve, purple). Thus, SAXS data do not provide any indication for a modification of the local nanostructure of the PAA-CB-PVA suspensions as compared to either PAA solutions or CB-PVA dispersions. Note that this is quite different from the case of PAA in PVA solutions, presented in Figure 4, where PAA-PVA interactions modify the scattering curve of the mixture.

## 4. Discussion

**Figure 7:**
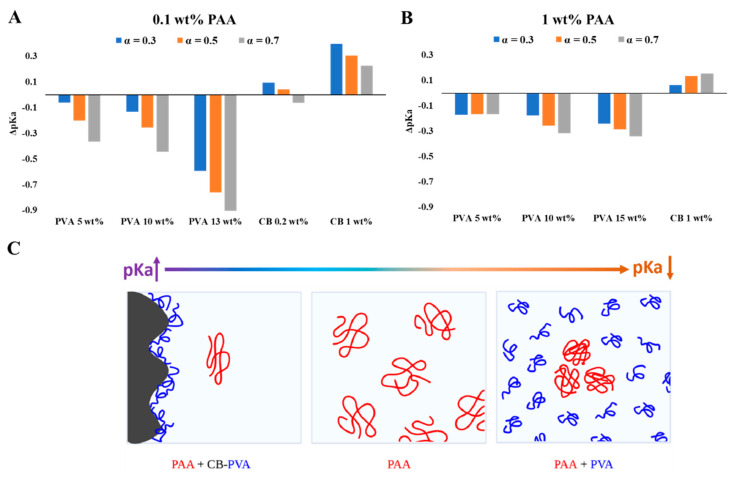
The $pK_{a}$ shift (compared to DIW) of PAA 100 kDa 0.1 wt% (**A**) and 1 wt% (**B**) at different ionization degrees. (**C**) Schematic illustration of the mixtures.

WPEs are responsive materials that modulate their ionization state by what is known as charge regulation (CR). Coupling between different degrees of freedom, and in particular between the acid–base reaction and the conformational degrees of freedom of the polymeric chains, is the origin of their ability to respond to environmental conditions and external constraints [24,25,26]. While different aspects of CR and chain conformations were investigated [27], here, we focus on the effect of non-charged, non-adsorbing macromolecules and colloidal particles on PAA, a fully hydrophilic, highly soluble WPE. Early studies of PAA have shown that chain connectivity reduces the fraction of charged monomers compared to monomeric acids due to coupling between charges located along the chain [5,28,29]. This is clearly observed in the results presented in Figure 1, where the degree of ionization of PAA is lower than that of Pac, a monomeric acid of a similar structure. In the following experiments, we measured titration curves of PAA (low concentrations (C<< C*), no added salt) in concentrated PVA solutions (C = 5, 10, ≤15 wt%). The dimensions of the relatively short-chain PVA and the high concentrations are relevant to bio-fluids where high concentrations of macromolecules and molecular assemblies are present [9,30,31]. The titration curves and the calculated $pK_{a}$ values for different degrees of ionization (Figure 2 and Figure 3) show significant deviations in the degree of ionization of PAA compared to those measured in water (Figure 1). We further used the very same PVA to disperse CB and investigated the titration curves of PAA in the resulting colloidal dispersions, where the typical dimensions of the additives are in the range of tens to hundreds of nanometers (see Figure 6A). The main results of this study, summarized in Figure 7, are the observed shift in the $pK_{a}$ of PAA due to solvated PVA and the presence of CB-PVA colloids. 

Our observations indicate that solvated (short) PVA chains and (large) PVA-decorated CB modify the acid–base equilibrium of the PAA in opposite directions: $pK_{a}$ increases in the presence of high concentrations of short PVA chains (indicating a higher degree of ionization than that measured in PVA-free solutions) and $pK_{a}$ decreases (a lower degree of ionization) in dispersions of CB-PVA (at low concentration of CB-PVA, as compared to PVA chains). In particular, we observe that at a given *pH*, the degree of ionization of PAA 100 kDa is higher in the presence of (the relatively short, $M_{w}$ = 13–23 kDa) PVA than in water. For example, for PAA (0.1 wt% PAA 100 kDa) at *pH* = 6, α = 0.39 in water, α = 0.43 in 10 wt% PVA solution, and α = 0.60 at 13 wt% PVA solution. In the colloidal dispersions of CB-PVA, the degree of ionization is lower by about 0.3 *pH* units (at low CB-PVA concentration, 0.2 wt%). Note that the presence of only 1 wt% of CB-PVA is enough to induce a deviation of similar magnitude as a PVA solution of 10 wt% (but in the opposite direction). 

To investigate the origins of this effect, we carried out a series of experiments. First, we investigated the effect of PVA on the titration curve of the monomeric acid, PAc, at concentrations of 0.1 and 1 wt% (Figure S2 of the ESI). It is known that PVA does not adsorb (or form complexes) with PAA [11], and thus no specific interaction with the functional group comprising both PAA and PAc (see Sketch 1) was expected. Indeed, no effect on the acid–base equilibrium of PAc was observed. We also prepared CB dispersions using Pluronic block-copolymers, F108, by following the procedure described in the experimental part (Figure S5 of the ESI). PAA titration of the CB-F108 dispersions exhibited similar behavior to that of CB-PVA, while the details of the interaction were complicated by the *pH*-dependent complexation of the protonated carboxylic group and the etheric oxygen of the EO group (see our previous report in ref [10]). 

Investigation of the nanostructure of solvated PAA in PVA solutions using SAXS revealed that the relatively small PVA chains (exhibiting a random coil conformation) induced what seems to be a crowding effect on the solvated PAA chains, leading to a (local) higher (by about 60%) concentration of PAA, and a consequential modification of the conformations of the PAA. On the other hand, scattering curves of PAA in the CB-PVA suspensions did not show such an effect, nor any effect of PAA on the aggregation of the CB-PVA nanoparticles. 

It is reasonable to assume that the effects observed in the PVA solutions resulted from crowding of the (relatively long) PAA chains by the (relatively short) PVA macromolecules [32]. Excluded volume and depletion interactions [33,34] induced by the colloidal CB-PVA dispersions probably modified the conformations of the PAA chains. Thus, the observed shift in the acid–base reaction probably resulted from entropic effects. Yet, rationalization of the detailed molecular mechanism leading to the reported results would require theoretical analysis and simulation studies that are beyond the scope of this study. 

To summarize, the findings presented here indicate that molecularly-induced crowding and excluded volume interactions, which are ubiquitous and fundamental features of any synthetic and bio-related multi-component fluids (again ref [9]), have a significant effect on the CR of WPEs.

## 5. Conclusions

The acid–base reaction of a model WPE is affected by the presence of molecular additives. High concentrations of non-ionic polymer and colloids modify the acid–base reaction, causing up to almost one *pH* unit shift in the $pK_{a}$.

Thus, when utilizing WPEs in bio-related fluids or synthetic complex fluid environments, one should take into account that coupling between the acid–base reaction of the polyelectrolytes and solvated macromolecules, nanoparticles, and colloids may shift the degree of charging of the WPEs, and consequentially their functionality and responsivity.

## Figures and Tables

**Table 1:** Polyacrylic acid properties in salt-free solutions [10].

$M_{w}$ [kDa]	N (Kuhn Segments)	R_H_ in Water [nm]	C* [wt%]	$pK_{a}$ (α=0.5)
30	314	4.7	4.51 ± 0.05	6.1
100	1047	9.6	2.31 ± 0.05	6.3

## Data Availability

The data presented in this study are available on request from the corresponding author.

## Supplementary Materials

The following supporting information can be downloaded at: https://www.mdpi.com/article/10.3390/polym15051121/s1, Figure S1: Degree of ionization as a function of *pH* in PAA solutions. PAA 100 kDa 1 wt% in DIW. Figure S2: PAc 0.1 wt% and 1 wt%, titration curve, and Δ$pK_{a}$ bar graph for ionization ratios of 0.3, 0.5, and 0.7. Figure S3: Viscosity measurements of PVA at different concentrations PVA 5–15 wt%. G’, G” plot of 15 wt% PVA. Viscosity of PVA 5 wt% + PAA 100 kDA 1 wt% at *pH* 3 and *pH* 7. Viscosity measurements of PAA 100 kDA 1 wt% at *pH* 3 and *pH* 7. Figure S4: Cryo-TEM of 1 wt% CB-PVA at *pH* = 4 and *pH* = 7. Figure S5: (A) Titration curves presenting the degree of ionization as a function of *pH*, $pK_{a}$ as function of a ^1/3^ and Δ$pK_{a}$ of PAA-CB dispersion at 30, 50, and 70% ionization, for PAA 100 kDa 0.1 wt% with CB-PVA and CB-F108. Figure S6: 1D SAXS curves obtained from 0.8 wt%, 1 wt%, 1.6 wt%, and 2 wt% PAA solution at *pH* = 7. Figure S7: SAXS curve obtained from 1 wt% PAA in 5 wt% PVA solution at *pH* = 7, and calculated curve via superposition of 1.6 wt% PAA and 5 wt% PVA at *pH* = 7. Table S1: Best fit parameters from the Lorentzian peak empirical model of the scattering curves of 0.8, 1, 1.6, and 2 wt% PAA 100 kDa. Table S2: Best fit parameters from the Lorentzian peak empirical model of the scattering curves of 1 wt% PAA 100 kDa at different *pH*. Table S3: Best fit parameters from the Orenstein–Zernike model of the scattering curves of 5 wt% PAA 100 kDa at different *pH*.
