# Supplementary material for: Charge Regulation of Poly(acrylic acid) in Solutions of Non-Charged Polymer and Colloids
Source: Polymers (Basel). 2023 Feb 23;15(5):1121. doi: 10.3390/polym15051121 (PMC10007678; doi:10.3390/polym15051121)
Supplement: Supplementary file 1 [file polymers-15-01121-s001.zip › polymers-2231502-supplementary.pdf]

ESI

Charge regulation of poly(acrylic acid) in solutions of non-charged polymer and colloids.

Evgenee Yekymov [1,†], David Attia [1,†], Yael Levi-Kalisman [2], Ronit Bitton [1,3] and Rachel Yerushalmi-Rozen [1,3,*]

[1] Department of Chemical Engineering, Ben-Gurion University of the Negev, 84105 Beer-Sheva, Israel.

[2] The Center for Nanoscience and Nanotechnology, and The Institute of Life Sciences at The Hebrew University of Jerusalem, Jerusalem 9190401, Israel.

[3] The Ilse Katz Institute for Nanoscience and Technology, Ben-Gurion University of the Negev, 84105 Beer-Sheva, Israel.

[†] These authors contributed equally to this work.

[*] Correspondence: rachely@bgu.ac.il.

In this study we investigated charge regulation (CR) due to non-specific interactions in a model system of a weak polyelectrolyte (WPE), poly(acrylic acid), PAA, in concentrated solutions of poly(vinyl alcohol), PVA, and colloidal dispersion of carbon-black dispersed by PVA (CB-PVA).

2. 1 Titration experiments, comparing between potentiometric titration and FTIR

The degree of ionization, α, of PAA can be measured via FTIR spectroscopy by linear superposition of the spectra obtained from hydrolyzed PAA at pH=2 and ionized PAA at pH=13 (see previous paper [1]). A comparison of titration curves obtained via potentiometric method and direct ATR-FTIR measurement is presented in Figure S1.

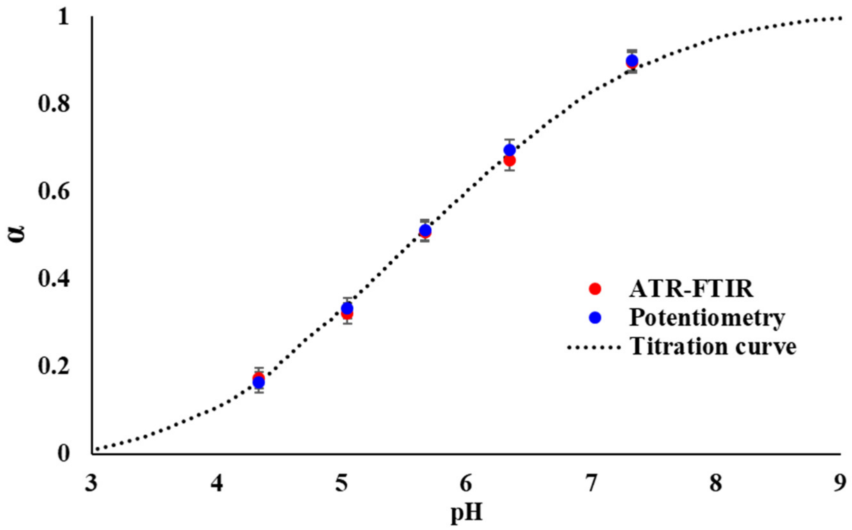

Figure S1. Degree of ionization as a function of pH in PAA solutions (PAA 100kDa, 1wt%, DIW no added salt). The dashed line is the calculated titration curve, blue dots were obtained from potentiometric measurements, red dots from FTIR measurements.

2.2 Calculation of the titration curves

98% hydrolyzed PVA 13-23 kDa contains less than ~1.55wt% of acetate group, with a monomeric pKa of 4.72 [2-4] similar to that of acetic acid.

The following calculation describes the ionization ratio of two weak acids A, and B, with a similar pKa but different concentrations of acidic groups.

$$(S1) \quad \text{HA} \leftrightarrow H^+ + A^- \quad and \quad \text{HA} \leftrightarrow H^+ + A^-$$

$$(A_0 - A' - a) \leftrightarrow (a + b) + (A' + a) \qquad (B_0 - B' - b) \leftrightarrow (a + b) + (B' + b)$$

Where A_0 and B_0 are the total acids concentration, A' and B' are the part of acid A and acid B reacted with the base during the titration. And a and b are part of acid A and acid B dissociated to reach the equilibrium (equation S2 and S3)

$$(S2) \quad \text{pKa}_\text{A} = \frac{[H^+][A' - a]}{[A_0 - A' - a]} = \frac{[10^{-pH}][y]}{[A_0 - y]}$$

$$(S3) \quad \text{pKa}_\text{B} = \frac{[H^+][B' - b]}{[B_0 - B' - b]} = \frac{[10^{-pH}][x]}{[B_0 - x]}$$

The pKa_A and pKa_B are dissociation constants for weak polyelectrolyte (A) and weak electrolyte (B). The pKa_B is constant throughout the titration. Thus, at any pH the x is:

$$(S4) \quad x = \frac{\text{pKa}_\text{B}\ \text{B}_0}{\text{B}_0 + 10^{-\text{pH}}}$$

And from charge conservation ($Na^+ \gg OH^-$)

$$(S5) \quad y = Na^+ + H^+ - OH^- - y \cong Na^+ + H^+ - x$$

Where Na^+ is concentration of the base added during the titration.

Thus the pKa_A of weak polyelectrolyte calculated via following formula:

$$(S6) \quad \text{pKa}_\text{A} = \frac{[10^{-pH}][y]}{[A_0 - y]} = \frac{[10^{-pH}]\left[Na^+ + H^+ - \frac{\text{pKa}_\text{B}\ \text{B}_0}{\text{B}_0 + 10^{-\text{pH}}}\right]}{\left[A_0 - Na^+ + H^+ - \frac{\text{pKa}_\text{B}\ \text{B}_0}{\text{B}_0 + 10^{-\text{pH}}}\right]}$$

The assumption leading to equation S6 is that pKa_B is constant.

In Figure S2 we present titration curves of PAc in the presence of PVA. The curves show that the degree of ionization of the PAc is similar in DIW and PVA solutions. The pKa values are not affected significantly by the presence of 5 wt% PVA 98% hydrolysed.

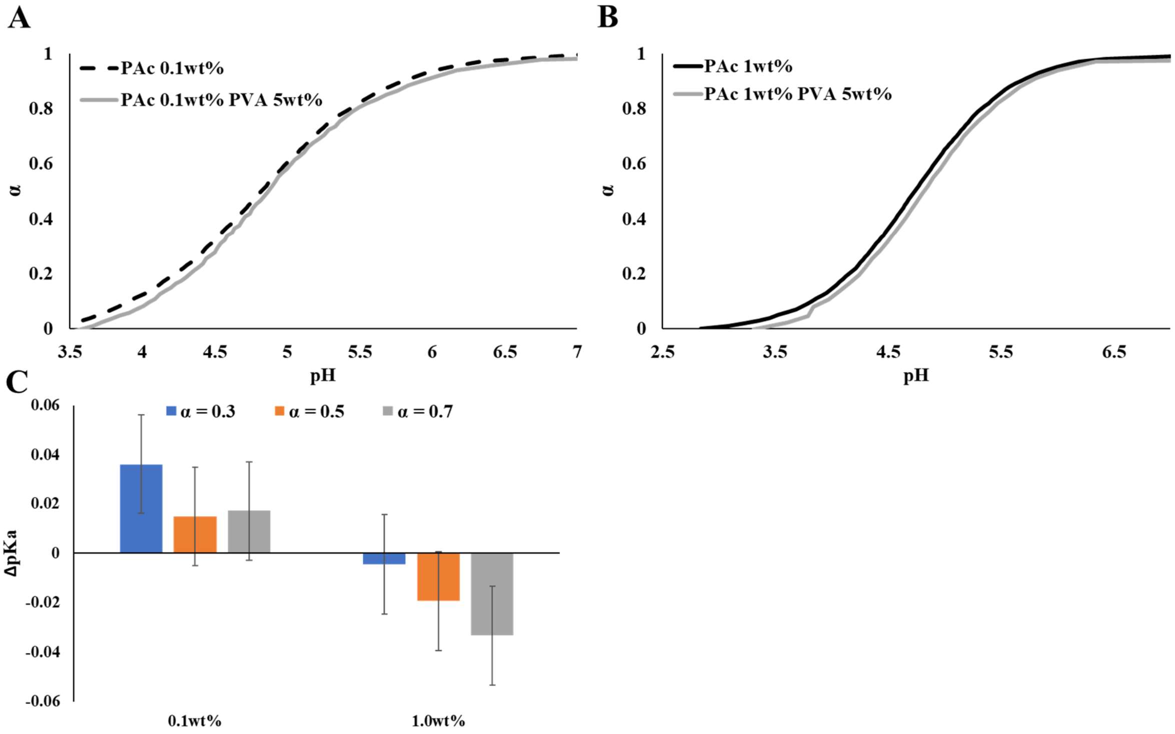

Figure S2. PAc titration curves (0.1wt% (A) and 1wt% (B)) and ΔpKa (C) bar graph for ionization degree of 0.3, 0.5 and 0.7. Black curves are DIW solution and grey are for PVA 5wt%.

3. Viscosity measurements of PVA solutions

The viscosity of PAA and PAA-PVA solutions was measured by MCR 702e MultiDrive rheometer (Anton Paar, Graz, Austria), operated in constant strain mode. Experiments of frequency, stress, and time sweeps were performed using 50 mm diameter parallel plates at 20 °C. Viscosity measurements were performed at shear rates between 1 and 1000 s^{-1} using 50 mm 1° steel cone geometry. Solvent evaporation was prevented by a solvent trap.

As shown in Figure S3, PVA solutions at concentrations below 15 wt% show Newtonian's behaviour. The G' and G" of PVA below 15wt% cannot be measured because of signal to noise limitations. Only at 15wt% PVA solution shows a viscoelastic behaviour and the G' and G" of PVA can be measured (Figure S3B).

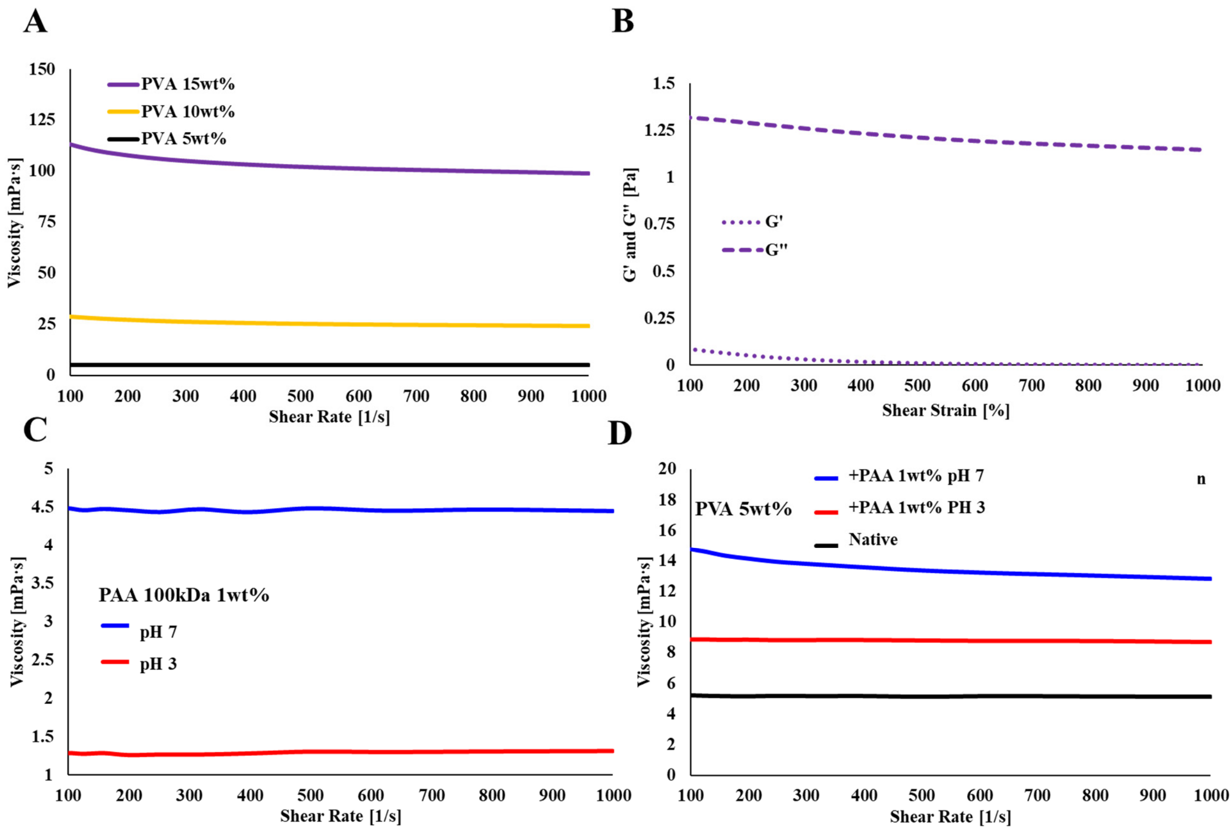

Figure S3. Viscosity measurements of PVA at different concentrations (A) PVA 5-15wt%. B) G', G" plot of 15 wt% PVA. C) Viscosity measurements of PAA 100kDA 1wt% at pH 3 and pH 7. D) Viscosity of PVA 5wt% + PAA 100kDA 1wt % at pH 3 and pH 7.

The viscosity of PAA solution (with and without PVA) increases with the pH [6] but remains Newtonian. Liquid mixtures of PVA-PAA show a similar behaviour to that of PVA solutions [5].

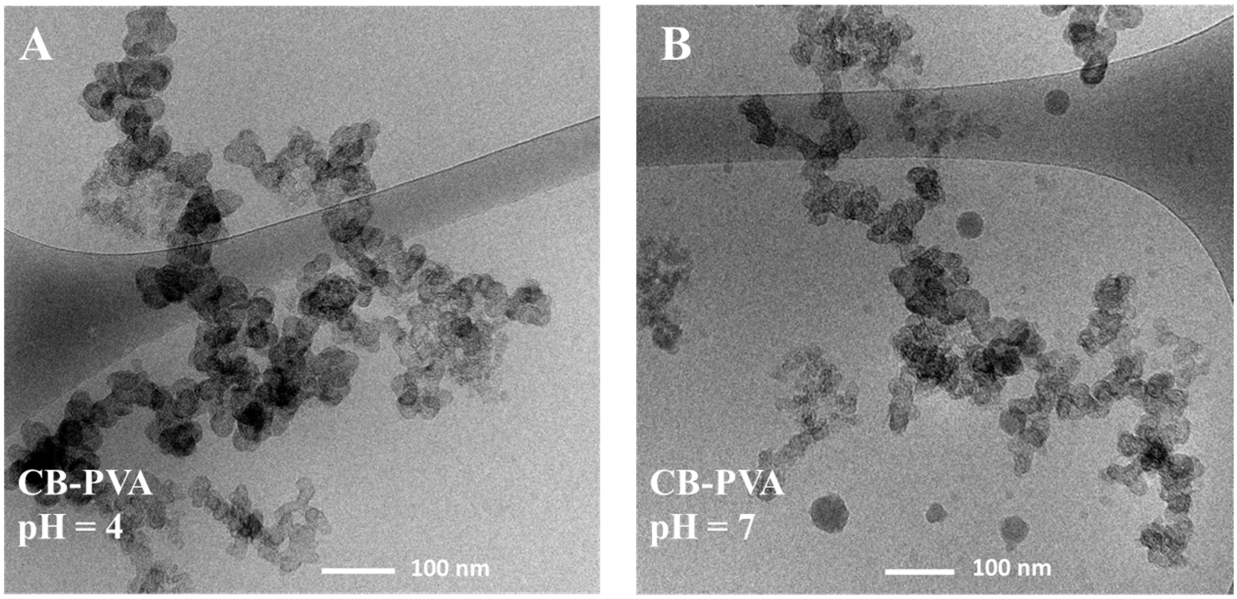

Figure S4. Cryo-TEM images of 1wt% CB-PVA at A) pH=4, and B) pH=7.

In Figure S4 we present cryo-TEM images of dispersions of CB-PVA in PAA solutions at pH = 4 and pH= 7. The images do not show a significant difference between the aggregation state of the CB-PVA particles.

4. Titrations of PAA in dispersions of CB

Materials: Carbon black nano powder (CB-Sigma product #699632), d < 500 nm) was purchased from Sigma Aldrich Israel. F108 (Pluronics poly (ethylene glycol)-*block*-poly(propylene glycol)-*block*-poly(ethylene glycol) polymers, $PEO_{100}PPO_{70}PEO_{100}$ was received as a gift from BASF (Product no. 583062).

Dispersions of CB in F108 solutions were prepared following the procedure described in the experimental part of the main text. Titrations experiments of PAA 100 kDa in CB-F108 dispersions are presented in Figure S5.

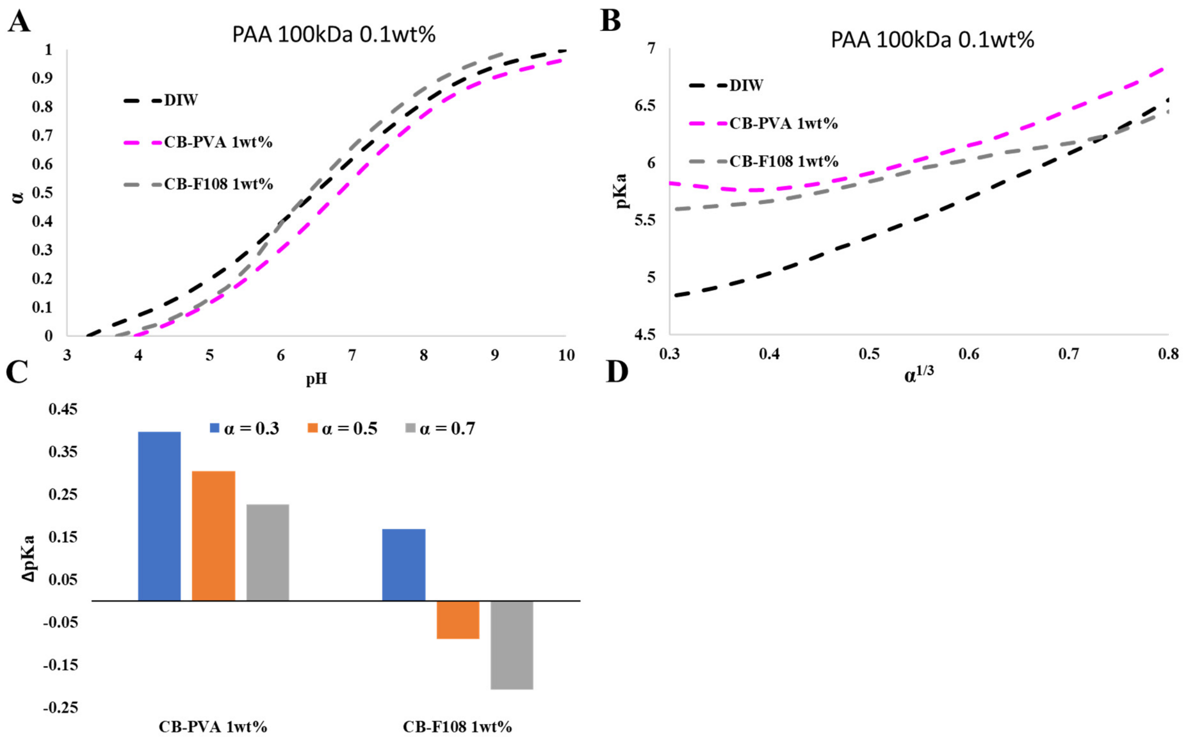

Figure S5. A) Titration curves presenting the degree of ionization (a) as a function of pH, (B) pKa as a function of a $^{1/3}$ and (C) ΔpKa of PAA-CB dispersion at 30, 50, and 70% of ionization, for PAA 100kDa 0.1wt% with CB-PVA and CB-F108.

The aim of these experiments was to investigate the role of the dimensions and geometry of the additives (CB) as compared to specific interactions induced by the dispersion polymer (PVA vs. F108). While PVA does not adsorb or bond to PAA (see main text and reference [7] where ITC measurements show weak interactions), PEO forms hydrogen bonding with the protonated acid comprising the PAA monomers [1], while it does not interact with the de-protonated (ionized) PAA. In Figure S5 C we observe deviations in pKa of magnitude similar to that observed in CB-PVA,

indicating that the degree of ionization along the titration curve is lower in both dispersions, irrespective of the difference in the specific interactions. A detailed description of the effect of micellar solutions on the acid-base reaction of PAA was given in our previous publication [1]. Indeed, for CB Sigma the shift is up to 0.6 pH units compared to 0.8 pH units for CB-PVA.

5. SAXS measurements

The scattering curves of PAA solutions (Figure 4A of the main text) are typical of polyelectrolyte solutions and exhibit the characteristic broad peak of charged PE [8]. As expected, the peak's position depends on the PAA concentration (Figure S6); measurements of PAA solutions at pH below (pH = 4) and above the pKa of PAA show that the peak appears due to ionization of the PAA and the location of the peak is not shifted by the pH (in accordance with previous reports, ref [9]).

SAXS curves obtained from solutions of PAA 100 kDa at different concentrations (pH = 7) are presented in Figure S6. All curves were fitted to the broad Lorentzian peak empirical model[10].

$$(S7) \qquad I(q) = \frac{A}{q^n} + \frac{C}{1 + (|q - q_0|\xi)^m}$$

Where A is the Porod law scale factor and n is the Porod exponent, C is the Lorentzian scale factor, ξ is the screening correlation length, and m is roughly equal to $1/\nu$ Where ν is the Flory exponent, which indicates the behavior of a polymer in a solvent (3/5 for good solvent, 1/2 for theta solvent)[11]. The peak maximum, q_0, corresponds to $d_0 = 2\pi/q_0$, representing an average distance between the charged PAA segments.

The best fit parameters from the Lorentzian peak empirical model correspond to Figure 4A and are summarized in Table S1.

Table S1. Best fit parameters from the Lorentzian peak empirical model of the scattering curves of 0.8, 1, 1.6 and 2 wt% PAA 100 kDa.

c [wt%]	A	n	C	q_0 [A^{-1}]	d_0 [nm]	ξ [nm]	m
0.8	0	0	2.91	0.049 ± 0.001	13 ± 1	2.5 ± 0.1	1.47 ± 0.03
1	0	0	3.17	0.060 ± 0.001	10 ± 1	2.0 ± 0.1	1.55 ± 0.02
1.6	0	0	2.99	0.069 ± 0.001	9 ± 1	2.0 ± 0.1	1.50 ± 0.04
2	0	0	1.27	0.075 ± 0.001	8 ± 1	1.8 ± 0.1	1.62 ± 0.02

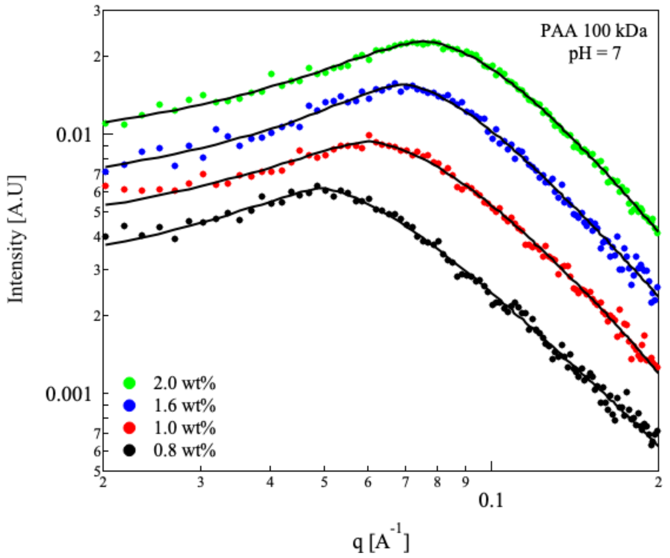

Figure S6. 1D SAXS curves obtained from 0.8 wt% (●), 1 wt% (●), 1.6 wt% (●), and 2 wt% (●) PAA solution at pH = 7. The solid lines correspond to the Lorentzian peak empirical model. The curves are shifted for better visualization.

The effect of different ionization degrees on the 1D scattering patterns obtained from solvated 1 wt% PAA 100 kDa was investigated using SAXS and presented in Figure 4 in the main text. The scattering patterns could be fitted to the Lorentzian peak empirical, and the best fit parameters to the model are summarized in Table S2.

Table S2. Best fit parameters from the Lorentzian peak empirical model of the scattering curves of 1 wt% PAA 100 kDa at different pH.

pH	*A*	*n*	*C*	q_0 [A^{-1}]	d_0 [nm]	ξ [nm]	*m*
4	0	0	17	0.033 ± 0.001	19 ± 1	2.5 ± 0.1	1.86 ± 0.01
5	0	0	17	0.050 ± 0.001	12 ± 1	2.5 ± 0.1	1.63 ± 0.01
5.6	0	0	19	0.054 ± 0.001	12 ± 1	2.5 ± 0.1	1.61 ± 0.01
7	0	0	26	0.053 ± 0.001	12 ± 1	2.4 ± 0.1	1.65 ± 0.01

* The parameters *A* and *n* correspond to the Porod upturn at low-q values of the Lorentzian peak empirical model. Since such an upturn was not observed in any of the SAXS curves obtained from PAA solutions, these parameters can be fixed as zero.

The scattering curves obtained from 5 wt% PVA solutions (Figure 4B) show the scattering typical to a random coil and could be fitted to the Ornstein-Zernike model[10].

$$(S8) \qquad I(q) = \frac{scale}{1 + (q\xi)^2} + B$$

Where ξ is the correlation length, and B is the background. The best fit parameters are summarized in Table S3.

Table S3. Best fit parameters from the Orenstein-Zernike model of the scattering curves of 5 wt% PAA 100 kDa at different pH.

pH	*ξ [nm]*
4	9.5 ± 0.1
7	9.7 ± 0.1

A comparison between a measured SAXS curve obtained from solvated 1 wt% PAA in 5 wt% PVA solution (pH = 7) and a calculated curve via linear superposition of 1.6 wt% PAA solution and 5 wt% PVA solution at pH = 7 is presented in Figure S7.

The black triangles (linear superposition of 1.6 wt% PAA solution and 5 wt% PVA solution at pH = 7) have a good fit to scattering of 1.0 wt% PAA solution and 5 wt% PVA solution at pH = 7. Therefore, we can conclude a local increase of PAA concentration following interaction with PVA. This observation presumably points to depletion interaction leading to local crowding of PAA.

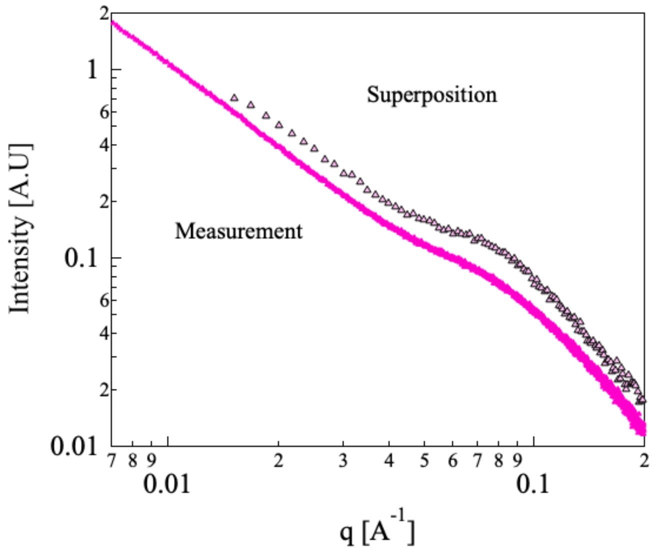

Figure S7. SAXS curve obtained from 1 wt% PAA in 5 wt% PVA solution at pH = 7 (▲), and calculated curve via superposition of 1.6 wt% PAA and 5 wt% PVA at pH = 7 (▲). The curves are shifted for better visualization.

References

1. Yekymov, E.; Attia, D.; Levi-Kalisman, Y.; Bitton, R.; Yerushalmi-Rozen, R. Effects of Non-Ionic Micelles on the Acid-Base Equilibria of a Weak Polyelectrolyte. *Polymers* **2022**, *14*, doi:10.3390/polym14091926.
2. Serjeant, E.P.; Dempsey, B. *Ionisation constants of organic acids in aqueous solution*; Pergamon: 1979; Volume 23.
3. Ouattara, B.; Simard, R.E.; Holley, R.A.; Piette, G.J.P.; Begin, A. Inhibitory effect of organic acids upon meat spoilage bacteria. *Journal of Food Protection* **1997**, *60*, 246-253, doi:10.4315/0362-028x-60.3.246.
4. Gildberg, A.; Raa, J. PROPERTIES OF A PROPIONIC ACID-FORMIC ACID PRESERVED SILAGE OF COD VISCERA. *Journal of the Science of Food and Agriculture* **1977**, *28*, 647-653, doi:10.1002/jsfa.2740280713.
5. Nagarkar, R.; Patel, J. Polyvinyl alcohol: A comprehensive study. *Acta Scientific Pharmaceutical Sciences* **2019**, *3*, 34-44.
6. Laguecir, A.; Ulrich, S.; Labille, J.; Fatin-Rouge, N.; Stoll, S.; Buffle, J. Size and pH effect on electrical and conformational behavior of poly(acrylic acid): Simulation and experiment. *European Polymer Journal* **2006**, *42*, 1135-1144, doi:10.1016/j.eurpolymj.2005.11.023.
7. Bizley, S.C.; Williams, A.C.; Khutoryanskiy, V.V. Thermodynamic and kinetic properties of interpolymer complexes assessed by isothermal titration calorimetry and surface plasmon resonance. *Soft Matter* **2014**, *10*, 8254-8260, doi:10.1039/c4sm01138d.
8. Horkay, F.; Hammouda, B. Small-angle neutron scattering from typical synthetic and biopolymer solutions. *Colloid and Polymer Science* **2008**, *286*, 611-620, doi:10.1007/s00396-008-1849-3.
9. Combet, J. Polyelectrolytes and small angle scattering. 2018; p. 03001.
10. Pedersen, J.S. Analysis of small-angle scattering data from colloids and polymer solutions: modeling and least-squares fitting. *Advances in Colloid and Interface Science* **1997**, *70*, 171-210, doi:10.1016/s0001-8686(97)00312-6.
11. Glatter, O. *Scattering methods and their application in colloid and interface science*; Elsevier: 2018.
